# Ceramide synthase *CERS4* gene downregulation is associated with *KRAS* mutation in colorectal cancer

**DOI:** 10.1038/s41598-023-43557-1

**Published:** 2023-09-27

**Authors:** Tamuro Hayama, Kotaro Hama, Tsuyoshi Ozawa, Yuko Fujiwara, Keijiro Nozawa, Keiji Matsuda, Kazuaki Yokoyama, Yojiro Hashiguchi, Hiroki Ochiai, Takeyuki Misawa, Takeo Fukagawa

**Affiliations:** 1 Department of Surgery, Teikyo University School of Medicine, 2-11-1 Kaga, Itabashi-ku, Tokyo, 173-8605 Japan; 2 Faculty of Pharma‑Sciences, Teikyo University, Tokyo, Japan; 3 Advanced Comprehensive Research Organization (ACRO), Teikyo University, Tokyo, Japan; 4 Kawaguchi the Institute of Proctology and Gastroenterology, Kawaguchi, Japan; 5 Department of Surgery, Japanese Red Cross Omori Hospital, Tokyo, Japan

**Keywords:** Tumour biomarkers, Cancer, Oncogenes

## Abstract

Ceramide, the central molecule in sphingolipid synthesis, is a bioactive lipid that serves as a regulatory molecule in the anti-inflammatory responses, apoptosis, programmed necrosis, autophagy, and cell motility of cancer cells. In particular, the authors have reported differences in sphingolipid content in colorectal cancer tissues. The associations among genetic mutations, clinicopathological factors, and sphingolipid metabolism in colorectal cancer (CRC) have not been investigated. The objective of this study is to investigate the association between genes associated with sphingolipid metabolism, genetic variations in colorectal cancer (CRC), and clinicopathological factors in CRC patients. We enrolled 82 consecutive patients with stage I–IV CRC who underwent tumor resection at a single institution in 2019–2021. We measured the expression levels of genes related to sphingolipid metabolism and examined the relationships between CRC gene mutations and the clinicopathological data of each individual patient. The relationship between CRC gene mutations and expression levels of ceramide synthase (*CERS*), *N*-acylsphingosine amidohydrolase (*ASAH)*, and alkaline ceramidase (*ACER*) genes involved in sphingolipid metabolism was examined CRES4 expression was significantly lower in the CRC *KRAS* gene mutation group (p = 0.004); vascular invasion was more common in colorectal cancer patients with high CERS4 expression (p = 0.0057). By examining the correlation between sphingolipid gene expression and clinical factors, we were able to identify cancer types in which sphingolipid metabolism is particularly relevant. *CERS4* expression was significantly reduced in *KRAS* mutant CRC. Moreover, CRC with decreased *CERS4* showed significantly more frequent venous invasion.

## Introduction

In recent years, it has been reported that ceramide plays an important role in ovarian cancer and lung cancer, and therapeutic agents to address impairments of the ceramide synthetic pathway are being developed^1,2^. Ceramide, a cell membrane lipid, and sphingosine-1 phosphate (S1P) are bioactive lipids in the sphingolipid synthesis pathway and play important roles in cell signaling. In vivo, they play opposite roles in cellular metabolism^3^. S1P stimulates cell survival, proliferation, and tissue regeneration, whereas ceramide has been shown to be involved in stress-related cellular responses and apoptosis^4,5^.

Apoptosis is associated with morphological changes at the membrane level of plasma and organelles, and the involvement of specific lipids in apoptosis has been established^6–8^. Maintaining a balance between ceramide and S1P is very important for cells as these bioactive lipids substantially contribute to cell fate determination. Alterations in ceramide levels have been recognized in pathological conditions such as inflammatory bowel disease, sepsis, and colon cancer^9,10^. Ceramide also plays important roles in regulating tumor growth and apoptosis; however, the mechanisms through which ceramide induces apoptosis are still unclear.

RAS is a group of four proteins involved in signal transduction that promotes cell proliferation. There are three RAS genes: *KRAS*, *NRAS*, and *HRAS*. Mutations in any of these genes produce abnormal RAS proteins. Point mutations in the RAS gene, particularly in the G12, G13, and Q61 hotspots, lead to a loss of GTPase activity, impaired GAP binding, and accumulation of active RAS protein and lead to activation of different intracellular signaling pathways. This aberrant signaling enhances proliferation and survival pathways in cancer cells, contributing to tumor development and progression across various types of carcinomas. It is thought that cancer can develop as a consequence due to signal transduction pathways that constantly promote cell proliferation^11^. It was reported that *KRAS* mutation colorectal cancer has a poorer prognosis than wild-type *KRAS* colorectal cancer^12,13^. One reason is that colorectal cancer with a *KRAS* mutation does not respond to cetuximab, whereas patients with colorectal cancer bearing wild-type *KRAS* benefited from cetuximab^14^. Elucidation of the mechanism of RAS mutation is very important due to its prominence as a marker for predicting the development and prognosis of new therapeutic agents. Our research focuses on establishing connections between the expression levels of sphingolipid-related genes expression levels and both *KRAS* mutations and various clinicopathological characteristics in patients diagnosed with colorectal cancer (CRC). We have chosen to investigate specific subtypes of CRC that are influenced by sphingolipid metabolism.

## Results

### Patient characteristics

Our study included a total of 82 patients. The median age was 68.0 (range 42–89) years, and 53 (64.6%) patients were male while 29 (35.4%) were female. The T factor (the depth of tumor invasion) was T1 or T2 in 11 (13.4%) and T3 or T4 in 71 (86.6%). There were 35 (42.7%) cases with lymph node metastasis (N factor+) and 47 (57.3%) cases without lymph node metastasis (N factor−). There were 36 (43.9%) cases with high preoperative CEA levels and 46 (56.1%) cases. There were 13 cases (15.9%) with distant metastases and 69 cases (84.1%) without. In terms of genes, *KRAS* mutations were found in 36 cases (43.9%), *NRAS* mutations were found in 2 cases (2.4%) (Table 1). *KRAS* mutations and *NRAS* mutations are detected mutations in exon 2, 3, and 4 regions of the respective genes.

**Table 1:** Clinicopathological features of the stage I–IV colorectal cancer patients who underwent tumor resection.

Variables	N = 82
Age (years), median	68.0
Gender (male/female)	53 (64.6)/29 (35.4)
Tumor location (right side/left side)	20 (24.4)/62 (75.6)
Histology (well or mod. defined/others)	79 (96.3)/3 (3.7)
Depth of tumor invasion (T1 or T2/T3 or T4)	11 (13.4)/71 (86.6)
Lymph node metastasis (+/−)	35 (42.7)/47 (57.3)
Lymph[atic] invasion (+/−)	41 (50.0)/41 (50.0)
Venous invasion (+/−)	60 (73.2)/22 (26.8)
Distant metastasis (+/−)	13 (15.9)/69 (74.1)
CEA [level] (high/normal)	36 (43.9)/46 (56.1)
KRAS (wild-type/mutant)	46 (56.1)/36 (43.9)
NRAS (wild-type/mutant)	80 (97.6)/2 (2.4)
BRAF (wild-type/mutant)	77 (93.9)/5 (6.1)

### Gene expression that contributes to ceramide in colorectal cancer

**Figure 1:**
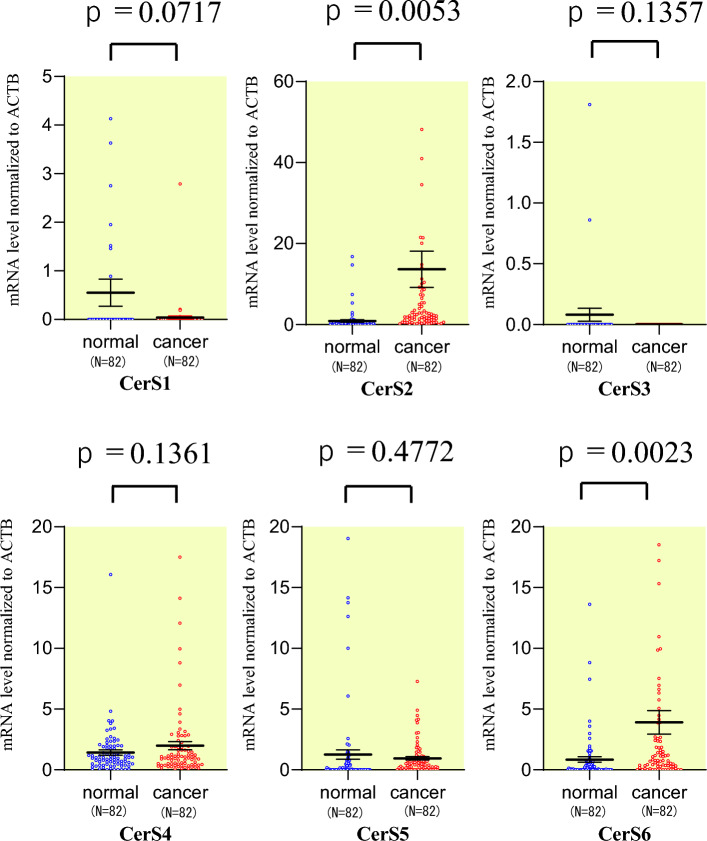
Expression levels of ceramide synthase (CERS) genes in normal tissues and colorectal cancer (CRC) tissues.

**Figure 2:**
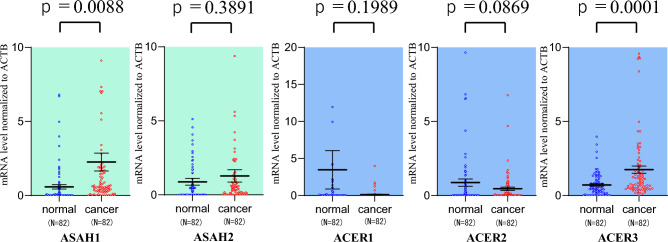
Expression level of *N*-acylsphingosine amidohydrolase (ASAH) and alkaline ceramidase (ACER) genes in normal tissues and colorectal cancer (CRC) tissues.

To investigate the role of *CERS1-6* in colorectal cancer, we examined the mRNA expression levels of *CERS1-6* in colorectal cancer tissue and paired normal colorectal mucosal tissue. The data generally showed significantly higher expression levels in CRC tissue than in normal tissue, with much higher mRNA relative levels of *CERS2* and *CERS6* (Fig. 1). Next, we investigated the gene expression of the enzymes that disassemble the fatty acid CoA, which is the substrate for *CERS*1–*CERS*6: namely, *ASAH1-2 and ACER1-3,* participating in the metabolic processes of sphingosine or ceramide. (Fig. 2). The results for mRNA expression levels of ASAH-1 and ACER3 in CRC tissue were significantly higher in cancer tissues than in normal paired tissue, respectively (p = 0.0088, 0.0001).

### Relationship between KRAS mutation and acyl-CoA synthase genes

**Figure 3:**
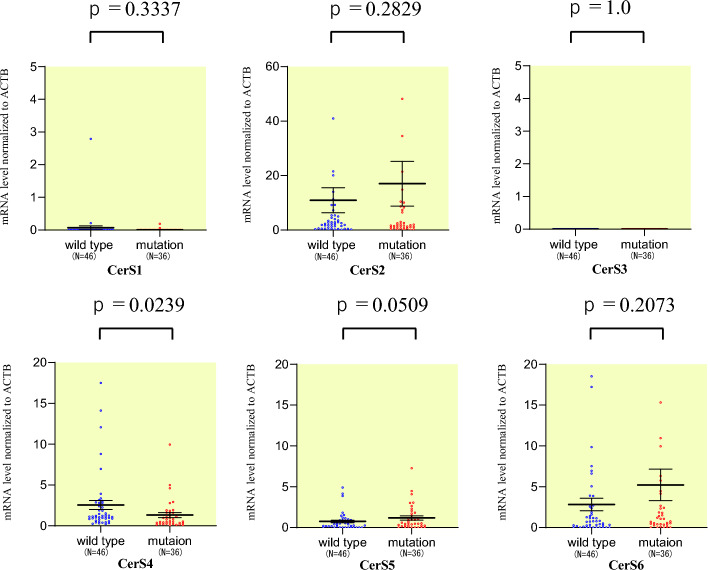
Quantities of *CERS4* in colorectal cancer tissue were compared for RAS gene status.

*CERS*, *ASAH*, and *ACER* gene expressions, which each contributes ceramide synthesis and degradation, were used to compare the characteristics of wild-type *KRAS* and *KRAS* mutant cancers. As a result, *CERS4* showed significantly low expression in *KRAS* mutant CRC (p = 0.004) (Table 2, Fig. 3).

**Table 2:** Relationship between KRAS status and the expression levels of genes involved in sphingolipid metabolism in colorectal cancer patients.

Variables	KRAS wild (n = 46)	KRAS mutation (n = 36)	p-value
CerS1	0.06, 0–2.79	0.0006, 0–0.18	0.3242
CerS2	16.1, 0.04–263.4	10.1, 0.0009–164.8	0.4726
CerS3	0	0	0.270
CerS4	2.71, 0.23–17.50	1.07, 0.07–4.99	0.004
CerS5	0.78, 0–4.91	1.16, 0–7.28	0.1827
CerS6	3.23, 0–23.5	4.78, 0–64.2	0.4238
ASAH1	2.67, 0–39.5	1.74, 0–16.7	0.4289
ASAH2	1.55, 0–32.4	0.92, 0–9.37	0.4225
ACER1	0.14, 0–3.99	0.07, 0–1.74	0.5116
ACER2	0.36, 0–2.35	0.58, 0–6.77	0.3048
ACER3	1.68, 0.20–9.58	1.82, 0–9.24	0.7794

### Cut-off value of CERS4 expression in KRAS mutation

**Figure 4:**
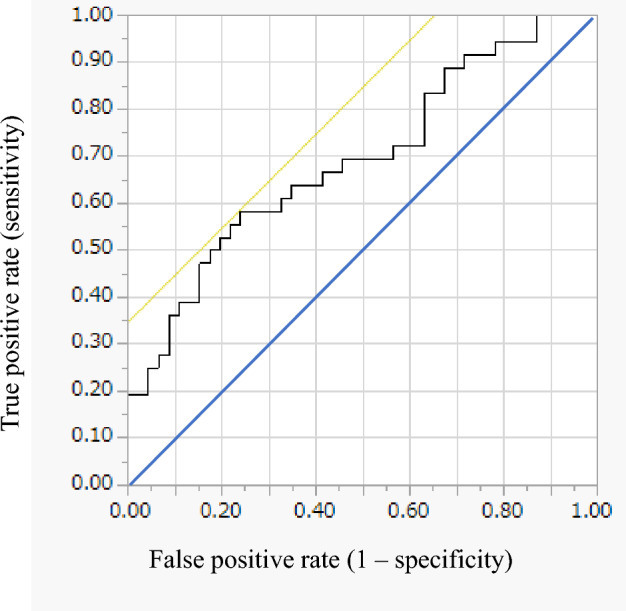
ROC for *CERS4* as a *KRAS* mutation factor for colorectal cancer was plotted to verify the optimum cutoff value of *CERS4*.

We performed ROC analyses to define the optimal cut-off value for *CERS4* expression in colorectal cancer tissue. ROC analyses showed that a *CERS4* cut-off value of 0.800 produced the best results, with an AUC of 0.694 (sensitivity: 0.83, specificity: 0.35) (Fig. 4).

### Comparison of low- and high-CERS4 groups

The target group was divided into two groups with the cut-off value of *CERS4* set to 0.80. *CERS4* < 0.8 was set as the low-*CERS4* group, and *CERS4* > 0.8 was set as the high-*CERS4* group. The two groups were compared for variables of age, gender, tumor location, histology, depth of tumor invasion, lymph node metastasis, lymph invasion, venous invasion, distant metastasis, CEA level, and *KRAS*, *NRAS*, and *HRAS* mutations. Venous invasion showed a significant difference, and venous invasion was significantly more prevalent in the low-*CERS4* group (p = 0.0057). There were significantly more cases of KRAS wild-type CRC in *high CERS4* group than in *low CERS4* group (p = 0.0015) (Table 3).

**Table 3:** Relationship between CERS4 status and clinicopathological factors in colorectal cancer patients.

Variables	CerS4 high N = 50	CerS4 low N = 32	p-value
Age (years), median	67.6	68.5	0.722
Gender (male/female)	31 (62%)/19 (38%)	22 (68.8%)/10 (31.3%)	0.533
Tumor location (right side/left side)	10 (20%)/40 (80%)	10 (31.3%)/22 (68.8%)	0.247
Histology (well or mod. defined/others)	48 (96%)/2 (4%)	31 (96.9%)/1 (3.1%)	0.837
Depth of tumor invasion (T1 or T2/T3 or T4)	5 (10%)/45 (90%)	6 (18.8%)/26 (81.3%)	0.257
Lymph node metastasis (+/−)	23 (46%)/27 (54%)	12 (37.5%)/20 (62.5%)	0.448
Lymph[atic] invasion (+/−)	19 (59.4%)/13 (40.6%)	22 (44%)/28 (56%)	0.1744
Venous invasion (+/−)	42 (84.0%)/8 (16%)	18 (56.3%)/14 (43.8%)	0.0057
Distant metastasis (+/−)	10 (20%)/40 (80%)	3 (9.4%)/29 (90.6%)	0.1998
CEA [level] (high/normal)	20 (40%)/30 (60%)	16 (50%)/16 (50%)	0.373
CA19-9 [level] (high/normal)	8 (16%)/42 (84%)	9 (28.1)/23 (71.9%)	0.1864
KRAS (wild-type/mutant)	35 (70%)/15 (30%)	11 (34.4%)/21 (65.6%)	0.0015
NRAS (wild-type/mutant)	32 (100%)/0 (0%)	48 (96.0%)/2 (4%)	0.2520
BRAF (wild-type/mutant)	29 (90.6%)/3 (9.4%)	48 (96%)/2 (4%)	0.3211

## Discussion

Currently, there is no information about the clinical significance of *CERS* genes in CRC progression. High expression levels of ceramide have been reported in patients with ovarian, lung and colorectal cancer^1,15,16^. In the present study, we comprehensively analyzed genes that contribute to ceramide synthesis and metabolism using colorectal cancer tissue. This study revealed two things. First, significant differences in gene expression between normal tissue and colorectal cancer tissue were observed for *ASAH1*, *ACER*3, *CERS*2, and *CERS*6. Second, we compared the expression of genes involved in ceramide synthesis and metabolism for *KRAS* mutations in CRC. It was revealed that *CERS4* expression was significantly lower in *KRAS* mutant CRC tissues compared with wild-type *KRAS* CRC tissues.

Several relationships between gene expression related to ceramide synthesis and metabolism and cancer sites have been reported. Zhang et al. reported that the expression level of *CERS*2 was decreased in highly metastatic ovarian cancer cells^1^. In ovarian cancer, Sheng et al. reported an association of high expression of *CERS*2 with an unfavorable prognosis in patients^17,18^.

Some reports have suggested that overexpressed *CERS6* promoted cancer invasion in lung cancer. They showed that overexpressed *CERS6* in lung cancer synthesizes a bioactive lipid called C16 ceramide, which activates the intracellular protein kinase, *RAC*1 complex. As a result, morphogenesis called lamellipodia, which is essential for cell migration, occurs on the cell surface, and cancer cells metastasize^2,16^.

In the present study, expression of *CERS4* was reduced in *KRAS* mutant colorectal cancer. There are two possible pathways for this result. The first is that the Wnt pathway signal is involved in the regulation of *CERS4* or the Wnt pathway may be involved in the *KRAS* mutation in colorectal cancer. Peters et al*.* used mice to show that *CERS4* is highly expressed in the epidermis of adult mice and is localized in defined populations within the interfollicular epidermis and hair follicle sebaceous unit. They reported that decreased bone morphogenetic protein signaling in *CERS4*−/− mice may promote Wnt/β-catenin signaling and strongly stimulate the activation of hair follicle stem cells^17^. In the present study, there are no data to positively demonstrate that the Wnt pathway is involved in the suppression of *CERS*4 in *KRAS* mutant CRCs, but this is a hypothesis.

The other possibility is that there is a relationship between *KRAS* mutations and the NF-kB (NF kappa B) pathway. NF-κB (NF kappa B) is a protein complex that acts as a transcription factor. Five types of proteins (NF-κB family) are known in mammals: p50, p52, p65 (RelA), c-Rel, and RelB^18,19^. It has been reported that p65 activity, which is one of the NF-KB family, was higher in tumors with *KRAS* mutation (50.8%) than in tumors with the wild-type *KRAS* gene (30.6%) (P = 0.012)^20^. In addition, there is a report that NF-KB activation is involved in *KRAS* mutant colorectal cancer, with NF-kB activity being higher than it is in wild-type *KRAS* colorectal cancer^21^. High expression of *CERS4* was observed in liver cancer tissues, and it has been reported that the nuclear factor (NF)-κB signaling pathway was affected after knockdown of *CERS4* in liver cancer cells^22^. Thus, even in *KRAS* mutant colorectal cancer, *CERS4* expression may be reduced due to the influence of the NF-kB pathway.

It has been reported that overexpression of *CERS4* and *CERS6* in colon cancer cells induced the production of short-chain ceramides (C16: 0, C18: 0 and C20: 0 ceramides), weakened cell proliferation and promoted apoptosis^23^. This is consistent with the poor prognosis of G12V and G12C mutation colorectal cancers we reported compared to wild-type *KRAS* colorectal cancers^12,13^. The present finding that *CERS4* functions as an important regulator of *KRAS* mutation, may suggest its potential use as a marker for CRC and may also guide the development of new drugs for CRC.

To assess the clinical attributes of individuals exhibiting elevated ceramide expression, we conducted a comparison of clinicopathological factors among CRC patients. Notably, the high *CERS*4 group displayed a higher prevalence of *KRAS* wild-type status and an increased incidence of positive venous invasion. It has been previously documented that *CERS4* and *CERS5* play a role in apoptosis within CRC^24^. Sphingosine 1-phosphate (S1P) is a blood-borne lipid mediator implicated in the regulation of vascular and immune systems. Blood flow and circulating S1P activate endothelial S1P1 to stabilize blood vessels in development and homeostasis^25^. Ceramides are apoptosis-inducing sphingolipids and precursors of other bioactive sphingolipids such as S1P. When ceramide is downregulated, its downstream S1P is also downregulated. This implies that *CERS*4 expression was reduced in the *KRAS* mutation group, potentially resulting in a diminished proapoptotic effect, may be why low-*CERS*4 cancers had more venous invasion.

This study’s limitation was that it included patients from just a single institution. Our study findings need further review and validation in more CRC patients.

## Conclusions

By examining the correlation between sphingolipids and clinical factors, we were able to identify cancer types in which sphingolipid metabolism is particularly relevant. Our study found that *CERS4* expression was significantly reduced in *KRAS* mutant CRC. Among the CRCs in which *CERS4* was decreased, there were significantly more cases with venous invasion than in the cases in which *CERS4* was not decreased.

## Patients and methods

### Patient selection

A total of 82 patients with stage I–IV CRC diagnosed based on the 8th edition of the United States Joint Commission on Cancer (AJCC)^26^ staging system and undergoing CRC resection at Teikyo University Hospital from 2019 to 2021 were enrolled in the study. This study was approved by Teikyo University ethics committee (Registration Number; 19-153). All participants provided informed consent, and our research findings are reported in adherence to the STROBE (Strengthening the Reporting of Observational Studies in Epidemiology) guidelines^27^. The experimental procedures conducted adhered to the ethical guidelines outlined in the Declaration of Helsinki, which is internationally recognized as the Code of Ethics for World Medicine.

### Sample preparation

All colorectal tissue specimens were dissected into small pieces, approximately 5 mm a side, frozen in liquid nitrogen within 24 h after resection, and stored at − 80 °C until lipid extraction. Normal tissues were obtained by lifting and dissecting the mucosa layer. The specimens mainly consisted of mucosa tissues but did not contain circular or longitudinal muscles.

### RAS and BRAF mutations analysis

Testing for *RAS/BRAF* mutations was performed at Hoken Kagaku Laboratories (Kanagawa, Japan) using samples collected from tumor tissues. The selected area of formalin-fixed paraffin-embedded samples (FFPEs) was deparaffined, followed by DNA isolation from samples using a QIAamp DNA FFPE Tissue Kit (Qiagen, Manchester, UK) according to the manufacturer’s instructions. DNA quantification was done on a NanoDrop 2000c (ThermoFisher Scientific, Waltham, MA, USA). The obtained DNA was amplified by a PCR reverse-sequence-specific oligonucleotide (PCR-rSSO) method on an Applied Biosystems VeritiTM 200 Thermal Cycler (Thermo Fisher Scientific) using MEBGENTM RASKET-B kit (MBL, Tokyo, Japan). Cycling began with 5 min at 40 °C and 2 min at 95 °C, followed by 10 cycles at 95 °C for 20 s and 62 °C for 30 s, then 45 cycles at 90 °C for 20 s, 60 °C for 30 s and 72 °C for 30 s. Finally, extension was performed for 1 min at 72 °C, followed by 1 min at 95 °C before the product was allowed to cool to 4 °C. The amplified PCR product was then hybridized with probes in Beads Mix in Hybridization Buffer provided with MEBGENTM RASKET-B kit. This reaction was done at 95 °C for 2 min followed by 55 °C for 20 min. The product was purified according to the manufacturer’s instructions and reacted with fluorescent phycoerythrin-labelled streptavidin. Fluorescence was measured by flow cytometry on a Luminex 100/200 System (Luminex, Austin, TX, USA), and the data were analyzed with the associated UniMAG software (Luminex).

### Quantitative real-time RT-PCR

Total RNA from colorectal tissues was extracted using an ISOGEN kit (Nippongene, Toyama, Japan), and cDNA libraries were synthesized using a shigh-capacity cDNA RT kit (Thermo Fisher Scientific). For cDNA synthesis, 2 µg of total RNA was used to yield 40 µL of reaction product. Then 1 µL of cDNA sample was used as a template per reaction for quantitative real-time PCR. We prepared cDNA samples from the 82 patients enrolled in this study and used them for quantitative real-time PCR. Due to shortages of cDNA samples for some patients, only 40 of the 82 patients were analyzed for SMS. The obtained results were normalized to the expression level of β-actin (*ACTB*) in each cDNA sample. The sequences of the oligonucleotides used in the PCR reaction were obtained from PrimerBank (https://pga.mgh.harvard.edu/primerbank/) or were originally designed and are listed as Table 4.

**Table 4:** List of oligonucleotide sequences used in the PCR reactions that were designed independently.

Gene	Sense (5′–3′)	Antisense (5′–3′)
*ACTB*	ATGAAGATCAAGATCATTGCTCCTC	ACATCTGCTGGAAGGTGGACA
*CERS1*	CCATGACCCACCATCTGTCT	TTGGTGAACTCAAGCTGCAC
*CERS2*	TCTATATCACGCTGCCCCTG	CTTGCCACTGGTCAGGTAGA
*CERS3*	ACTGTTGCTGGAATTGCGTT	TGTTACAGGTCTGCGTCCAT
*CERS4*	TCTCTGGTGCTGCTGTTACA	AGATGAGGAAGAGAGCGTCG
*CERS5*	CATCTTCTTCGTGAGGCTGC	TGTCCTGATTCCTCCGATGG
*CERS6*	ACCTGAAGAACACGGAGGAG	CCAGCAATGCCTCGTATTCC
*ASAH1*	TCAGGACCAACGTACAGAGG	AAAAGGGCCAGGAAAGTTGC
*ASAH2*	TGCACAGGACAAGTAGCAGA	CCATGATGAAGGCACGACTG
*ACER1*	CCTATCAGAGCTCCGAGGTG	AGTGGCCCGAAGATGAAGAA
*ACER2*	TCTCTCTGATGACCCTGGGA	GAGGCAGCATCAAAGTAGGC
*ACER3*	ACTGGTGCGAGGAGAACTAC	GAAGCACCAGGATCCCATTC
*SMS1*	GCATATTACATCACCACGAGACTCTT	GGAGGTTCATCTGGGAAGCTT
*SMS2*	TTCAGCGGTCACACGGTTAC	ACGAGGCGAATATTCTTTGATGA

### Statistical analysis

Those features that were found significant were further investigated to discover their predictive capability. To evaluate the diagnostic power of the devised panel of genes responsible for sphingolipid metabolism, receiver operating characteristic curves (ROC) were constructed, the area under the curve (AUC) was calculated, and the optimal cut-off values and Youden indexes were determined. Statistical significance was assumed when p-values were less than 0.05. All statistical analyses were performed using JMP 15 software (SAS, Cary, NC, USA).

### Ethics approval

This study was approved by the Teikyo University ethics committee (Registration Number; 19-153).

## Data Availability

The datasets collected in this study can be obtained from the corresponding author upon a reasonable request**.**
